# Effectiveness and safety of high dose clopidogrel plus aspirin in ischemic stroke patients with the single *CYP2C19* loss-of-function allele: a randomized trial

**DOI:** 10.1186/s12883-020-01974-z

**Published:** 2020-10-29

**Authors:** Hongliang Wu, Huiqun Song, Lianwei Dou, Bo Gao, Yan Pan, Mei Dong, Qi Chen, Jiazhen Li, Lixiang Song, Chuanyu Liu, Bing Li, Wenzheng Chu

**Affiliations:** 1 Department of Neurology, The Affiliated Yantai Yuhuangding Hospital of Qingdao University, Yantai, Shandong 264000 P.R. China; 2 Department of Radiology, The Affiliated Yantai Yuhuangding Hospital of Qingdao University, Yantai, Shandong 264000 P.R. China; 3 Department of Ultrasound, The Affiliated Yantai Yuhuangding Hospital of Qingdao University, Yantai, Shandong 264000 P.R. China; 4 Department of Cardiology, The Affiliated Yantai Yuhuangding Hospital of Qingdao University, Yantai, Shandong 264000 P.R. China

**Keywords:** Ischaemic stroke, Intracranial stenosis, Carotid stenosis, *CYP2C19*, Clopidogrel, Dual antiplatelet aggregation therapy

## Abstract

**Background:**

Dual antiplatelet aggregation therapy leads to better outcomes in patients with carotid artery stenosis, intracranial artery stenosis, minor strokes, or transient ischaemic attacks. However, carriers of the *CYP2C19* loss-of-function allele may not experience the desired effects. We attempted to increase the clopidogrel dose to determine whether it would improve the outcomes of stroke patients who carry a single loss-of-function allele.

**Methods:**

We recruited 131 patients with minor ischaemic stroke, within less than 7 days of stroke onset and a *CYP2C19* loss-of-function allele, who had moderate-to-severe cerebral artery stenosis. Patients were divided into the high dose group (clopidogrel 150 mg per day + aspirin 100 mg per day over 21 days.) and a normal dose group (clopidogrel 75 mg per day + aspirin 100 mg per day over 21 days). The reported outcomes included any vascular or major bleeding events as the primary and safety endpoints, respectively.

**Results:**

One and six vascular events occurred in the high dose and normal dose groups during the 3-months follow-up period, respectively. However, no significant difference was found between the two groups when adjusted for history of diabetes (hazard ratio, 5482; 95% confidence interval, 0.660 to 45.543; *P* = 0.115). No major bleeding events occurred.

**Conclusions:**

In patients with ischaemic stroke who had a single *CYP2C19* loss-of-function allele and moderate to severe cerebral stenosis, fewer vascular events occurred within 3 months with high dose of clopidogrel and aspirin than with normal dose of clopidogrel and aspirin. However, the difference between the two groups was not significant.

**Trial registration:**

Clinical study of clopidogrel in the treatment of patients with symptomatic moderate to severe cerebral artery stenosis with intermediate metabolites of *CYP2C19*, URL: http://www.chictr.org.cn/. Unique identifier: ChiCTR1800017411, 07/28/2018;

## Background

Stenosis of the cerebral blood supply arteries, including intracranial atherosclerotic stenosis or extracranial carotid stenosis, is a major contributing factor to ischaemic stroke and stroke recurrence. Several studies have shown that dual antiplatelet aggregation therapy is an effective treatment. Patients in the SAMMPRIS trial [1] achieved a better prognosis than those in the WASID trial, with aspirin and clopidogrel for 90 consecutive days while actively controlling lipid, blood pressure, and glucose levels. In another study [2], patients with intracranial artery stenosis who received double antiplatelet aggregation therapy with aspirin and clopidogrel within less than 7 days of onset were significantly less likely to have a microembolic signal detected on transcranial Doppler (TCD) than those who were administered only aspirin. In a study of symptomatic carotid stenosis [3], co-administration of clopidogrel and aspirin significantly reduced the occurrence of symptomatic embolism compared to the administration of only aspirin. In a trial of transient cerebral ischaemia and minor stroke [4], co-administration of clopidogrel and aspirin reduced the risk of recurrent stroke within 90 days compared to the administration of only aspirin, without increasing the risk of bleeding. However, in this trial, dual antiplatelet aggregation was superior to aspirin alone only in patients without *CYP2C19* loss-of-function allele (LoFA), which was present in 58.8% of patients [5].

Clopidogrel is biotransformed by cytochrome P-450 enzymes into an active metabolite. Patients carrying *CYP2C19* LoFAs have lower levels of clopidogrel active metabolites [6]. In East Asian populations, the most common LoFAs are CYP2C19*2 (frequency of 30–50%) and CYP2C19*3 (frequency of 5–10%) [7]. Carriers of these two LoFAs have a decreased platelet response to clopidogrel and an elevated cardiovascular disease risk [8–11]. Some studies have attempted to increase clopidogrel doses to treat ischaemic disease. In a trial of patients with acute coronary syndrome who were being prepared for percutaneous coronary intervention (PCI), randomization to double-dose clopidogrel (150 mg per day) had a lower risk of cardiovascular events and in-stent thrombosis than the standard dose (75 mg per day). This treatment increased the risk of bleeding, but not of fatal bleeding or intracranial hemorrhage [12]. This study did not select patients based on metabolic genes, which may have contributed to increased bleeding. In another trial [13], triple-dose clopidogrel (225 mg per day) enabled CYP2C19*2 heterozygotes to achieve platelet responsiveness at the level of normal doses (75 mg per day) for non-carriers. In contrast, for CYP2C19*2 homozygotes, the corresponding level of platelet inhibition was not achieved even at 300 mg/day. We speculate that for *CYP2C19* *1/*1 and *1/*17 carriers, higher doses of clopidogrel may lead to increased bleeding, whereas for two LoFA carriers, higher doses do not improve antiplatelet effects. In addition, Kobayashi et al. found that among healthy Japanese subjects, intermediate metabolic subjects who were administered a 7-day double dose of clopidogrel achieved an antiplatelet effect close to that of fast metabolic subjects and was well tolerated [14].

Based on these results, we selected patients with moderately severe cerebrovascular stenosis within less than 7 days of cerebral infarction onset, who had only one *CYP2C19* LoFA to understand the effectiveness and safety of double-dose clopidogrel administration.

Our study adheres to CONSORT guidelines.

## Methods

This is a prospective, single-centre, parallel-group, randomized, superiority trial. The study protocol was approved by the Ethics Committee of Yantai Yuhuangding Hospital (approval no. 193: [2017]). All patients provided informed consent prior to enrolment.

### Study population

#### Inclusion criteria

Patients with acute ischaemic stroke who are continuously hospitalised; aged ≥40 years and ≤ 75 years; with moderate to severe cerebral artery stenosis (stenosis > 50%) within less than 7 days of ischaemic stroke onset and access to the study drug within 24 h of admission; patients with a single *CYP2C19* LoFA (*1/*2, *1/*3).

After trial commencement, we added National Institutes of Health Stroke Scale (NIHSS) score ≤ 5 as an inclusion criterion to decrease the risk of cerebral haemorrhage.

#### Exclusion criteria

The present attack was confirmed as a non-cerebrovascular attack by cranial magnetic resonance imaging or cranial computed tomography; significant signs of anticoagulation were present (suspected cardiogenic embolism, such as rheumatic heart valve disease, known artificial heart valves, atrial fibrillation, and suspected endocarditis); bleeding from the gastrointestinal tract within 1 year, or positive faecal occult blood on admission to hospital; previous history of intracranial haemorrhage (cerebral haemorrhage or subarachnoid haemorrhage); severe heart failure, asthma, liver, and kidney insufficiency; previous history of coagulation abnormalities or systemic bleeding disorders, previous history of hemocytopenia, leukopoenia (< 2 × 10^9^/L), or thrombocytopenia (< 100 × 10^9^/L); patients who were given aspirin combined with clopidogrel therapy at randomisation; patients who were pregnant or participating in other clinical trials; and any patients or their legal representative who refused to participate in the investigation.

### Determination of arterial stenosis

Extracranial carotid artery (including internal and common carotid arteries) stenosis was determined by carotid Doppler sonography studies, which were performed by the same diagnostic sonographer in our hospital’s ultrasound department. The degree of stenosis was determined by a combined definition of peak systolic velocities, end-diastolic velocities, and B-mode [15].

Intracranial artery stenosis was determined by head magnetic resonance angiography (MRA). All MRAs were analyzed by the same diagnostic imaging physician. The degree of stenosis is determined by the narrowest diameter of the stenosis versus the normal vessel diameter of the same artery. The vessels included in this study were: the internal carotid artery intracranial segment, the middle cerebral artery M1 segment, the vertebral artery intracranial segment, and the basilar artery.

Those with presence of moderate to severe stenosis (≥50%) in the above vessels and infarct lesion located in the blood supply area of the stenotic vessel were eligible for inclusion in this study.

### Clinical data collection

We collected baseline data including: demographic data, past disease history, blood pressure, blood cell counts, fasting blood glucose, lipid series, bilirubin, transaminases, creatinine, urea, homocysteine, glycosylated haemoglobin, and uric acid. We routinely evaluated patients using the modified Rankin Scale (MRS) and the NIHSS score on admission and discharge.

We collected the above data from the hospital’s medical records system at patients’ discharge time.

### Treatment

Eligible patients were randomly assigned to treatment groups in a 1:1 ratio. The high dose group was administered 150 mg of clopidogrel and 100 mg of aspirin per day after initially receiving 300 mg of clopidogrel on day 1, while the normal dose group was given 75 mg of clopidogrel and 100 mg of aspirin per day after initially receiving 300 mg of clopidogrel on day 1. All patients were maintained on dual antiplatelet aggregation therapy for 21 days according to the two different regimens; then clopidogrel was discontinued, and aspirin 100 mg daily was continued throughout the observation period (90 days from stroke onset).

All patients were administered pantoprazole 40 mg per day during dual antiplatelet therapy and atorvastatin 20 mg per day throughout the observation period (90 days from stroke onset).

### Sample size and randomization

No previous studies have provided information on the incidence of vascular events within 90 days of mild stroke associated with cerebral stenosis. We estimated the incidence of vascular events in the control group based on data from previous trials [1, 4] to be greater than 20%, while the incidence in the high dose group was less than 5%. Taking the alpha value of 0.05, We decided to recruit at least 130 patients in total. We applied the simple randomization method to divide the recruited patients into the high dose group and the normal dose group in a 1:1 ratio.

### Endpoint

Information regarding vascular events during the follow-up was obtained for all patients through standard telephone interviews. The primary endpoint for the trial was the time from randomisation to the first vascular event within 3 months of the follow-up. A vascular event was defined as any stroke (including ischaemic or haemorrhagic), myocardial infarction, or death. The secondary endpoint was the neurological outcome at discharge, as assessed by the NIHSS score.

### Safety evaluation

The safety endpoint was defined as the time from randomization to a major bleeding event or any bleeding event that resulted in the trial treatment being discontinued, such as intracranial haemorrhage, gastrointestinal haemorrhage, haemoptysis, and pericardial occlusion.

### Statistical analysis

Baseline characteristics were compared between the high dose and normal dose groups. Categorical variables were displayed as percentages, and chi-square tests were used. Normally distributed continuous variables were presented as mean ± standard deviation and compared using analysis of variance (ANOVA). Continuous variables with abnormal distributions were presented as medians (interquartile ranges) and compared using the nonparametric Wilcoxon test between groups. The NIHSS scores were compared between the two groups by the nonparametric Wilcoxon test. Survival plots were generated by applying the Kaplan-Meier product limit method, and differences between treatment groups were tested for significance by the log-rank test. Cox regression models were used to assess the associations between treatment groups and vascular events during the 90-day follow-up period and adjusted based on the history of diabetes mellitus. All statistical analyses were performed using STATA (version 14.0; StataCorp LLC, College Station, TX, USA).

## Results

**Fig. 1:**
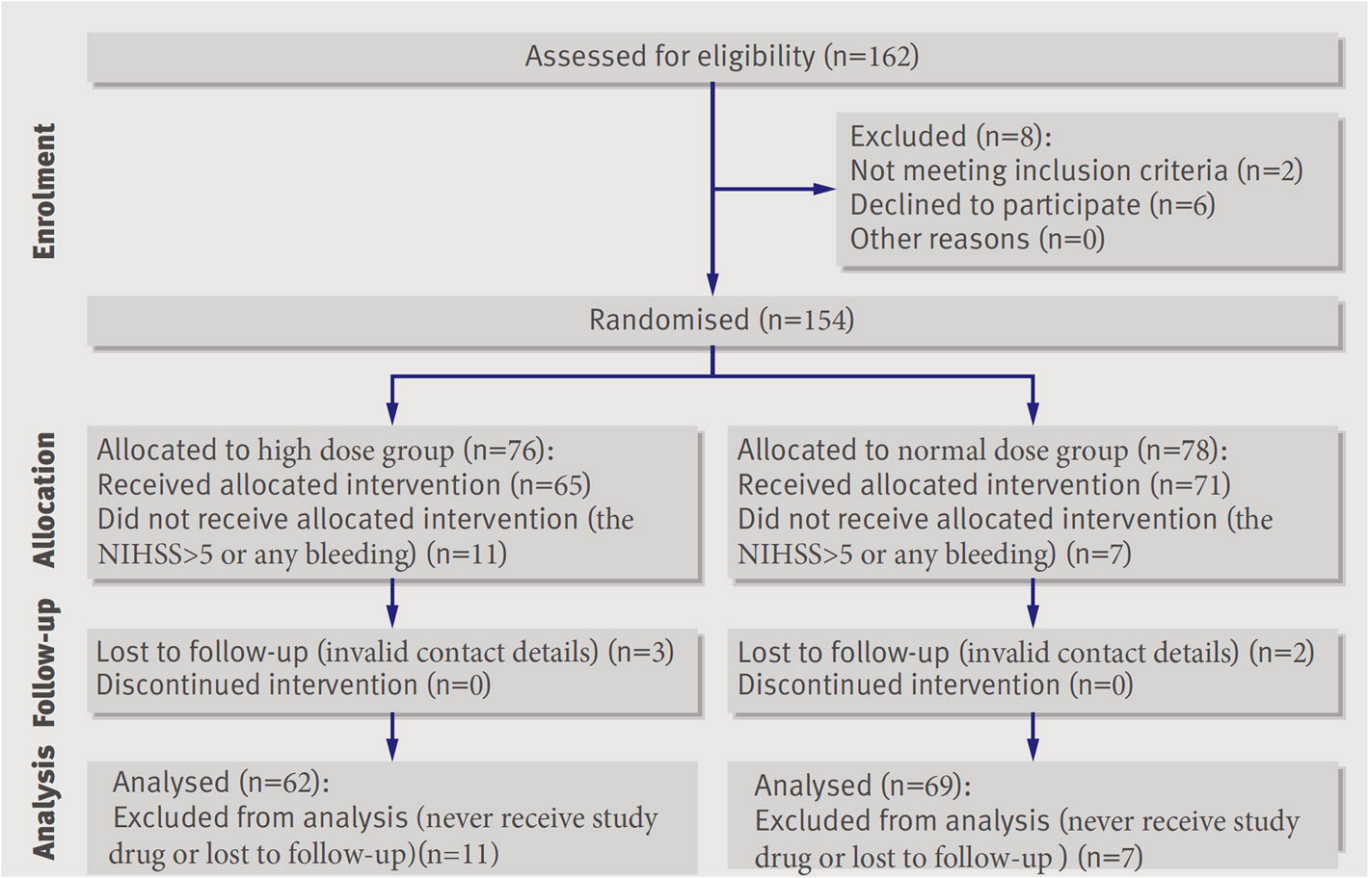
Study flow diagram

Overall, 162 patients were enrolled in this study from August 2018 to February 2019 and were followed up for a 90-day period, 154 patients underwent randomization, with 76 being placed in the high dose group and 78 in the normal dose group (Fig. 1). A total of 18 patients did not receive the trial intervention because of NIHSS > 5 or bleeding from any part of the body; 11 in the high dose group, and 7 in the normal dose group. Five patients were lost during follow-up because of invalid contact information, 3 in the high dose group, and 2 in the normal dose group. Ultimately, 131 patients were included in the analysis. Sixty-two patients were finally included in the high dose group and 69 in the normal dose group. Table 1 shows the baseline characteristics between the high dose and normal dose groups. The proportion of patients with a history of diabetes was significantly higher in the normal dose group than in the high dose group, but no difference in fasting glucose or glycosylated haemoglobin was observed between the two groups. Other baseline data such as sex, age, blood pressure, lipids, haemoglobin, and platelets did not differ between the two groups.

**Table 1:** Comparison of the baseline data between the two groups of patients

	High dose group	Normal dose group	*p*-value
Gender, n (male/%)	49 (79.03)	50 (72.46)	0.381
H. Stroke, n (%)	12 (19.35)	11 (15.94)	0.608
CHD, n (%)	7 (11.29)	8 (11.59)	0.956
DM, n (%)	18 (29.03)	32 (46.38)	0.040
Hypertension, n (%)	44 (70.97)	49 (71.01)	0.995
Smoker, n (%)	31 (50.00)	33 (47.83)	0.804
Drinker, n (%)	26 (41.94)	28 (40.58)	0.875
Age, median (IQR)	60.0 ± 10.4	63.2 ± 9.3	0.406
SBP, mmHg ± SD	148 (133–162)	151 (139–167)	0.390
DBP, mmHg ± SD	88 (78–95)	86 (79–96)	0.854
FBG, mmol/L, median (IQR)	5.18 (4.49–6.95)	5.54 (4.67–7.59)	0.178
GH, %, median (IQR)	5.9 (5.6–6.9)	6.1 (5.6–8.0)	0.409
HCY, umol/L, median (IQR)	12.1 (10.5–13.9)	11.9 (10.5–14.5)	0.921
PLT, ×10^9^/L, median (IQR)	229 (199–254)	217 (180–235)	0.402
HGB, g/L, median (IQR)	148 (140–158)	141 (132–153)	0.423
CHL, mmol/L, median (IQR)	4.27 (3.69–5.43)	4.02 (3.33–4.82)	0.332
HDL, mmol/L, median (IQR)	1.09 (0.94–1.26)	1.11 (0.92–1.27)	0.747
LDL, mmol/L, median (IQR)	2.47 (1.89–3.22)	2.21 (1.75–2.86)	0.970
TG, mmol/L, median (IQR)	1.28 (0.98–1.86)	1.30 (0.96–1.55)	0.627
AST U/L, median (IQR)	20.0 (16.0–26.0)	19.0 (16.0–25.0)	0.943
CRE, umol/L, median (IQR)	69.5 (57.0–77.0)	67.0 (55.0–84.0)	0.655
URE, mmol/L, median (IQR)	5.15 (4.34–6.07)	4.70 (4.04–5.74)	0.211
UA, umol/L, median (IQR)	318.1 ± 89.7	311.1 ± 79.7	0.341

*H. stroke* History of stroke, *CHD* Coronary atherosclerotic heart disease, *DM* History of diabetes mellitus, *SBP* Systolic blood pressure, *DBP* Diastolic blood pressure, *FBG* Fasting blood glucose, *GH* Glycosylated haemoglobin, *HCY* Homocysteine, *PLT* Platelets, *HGB* Haemoglobin, *CHL* Cholesterol, *HDL* High-density lipoprotein, *LDL* Low-density lipoprotein, *TG* Triglyceride, *AST* Aspartate aminotransferase, *CRE* Creatinine, *URE* Urea, *UA* Uric acid

**Fig. 2:**
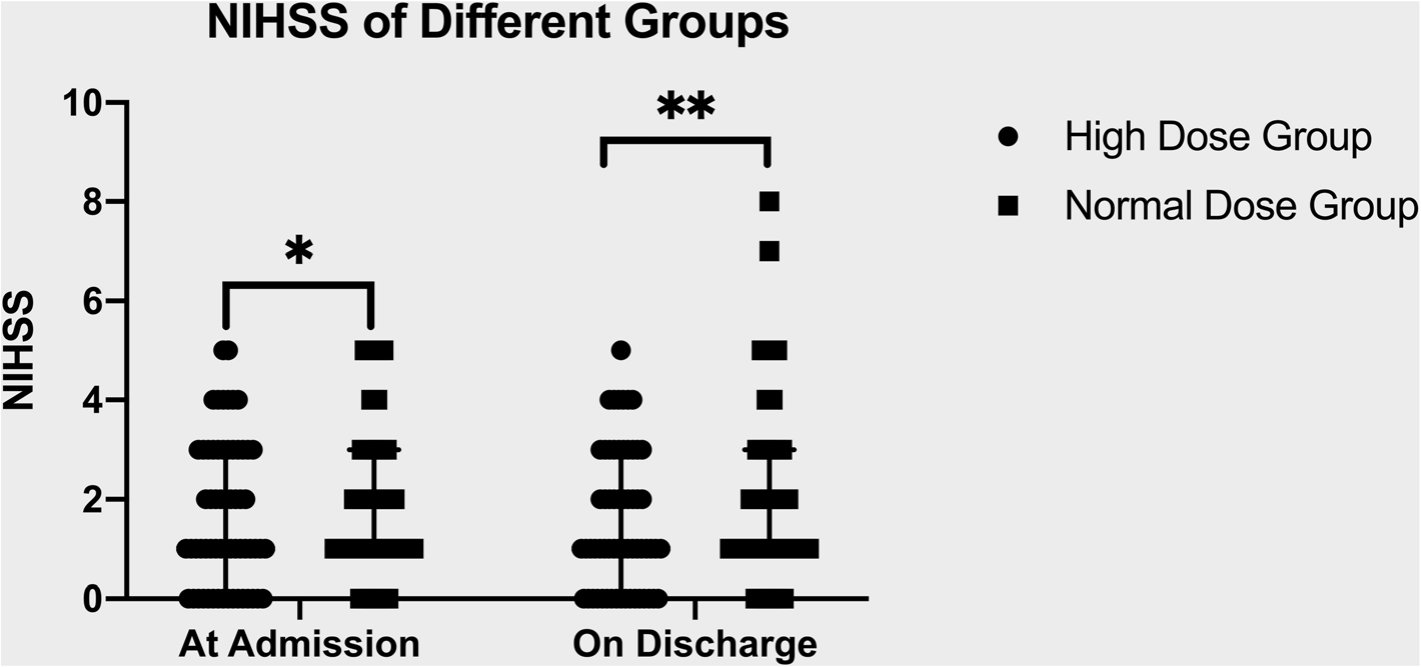
Mann-Whitney test, *p = 0.5286 ** *p* = 0.2192. NIHSS indicates National Institutes of Health stroke scale

**Fig. 3:**
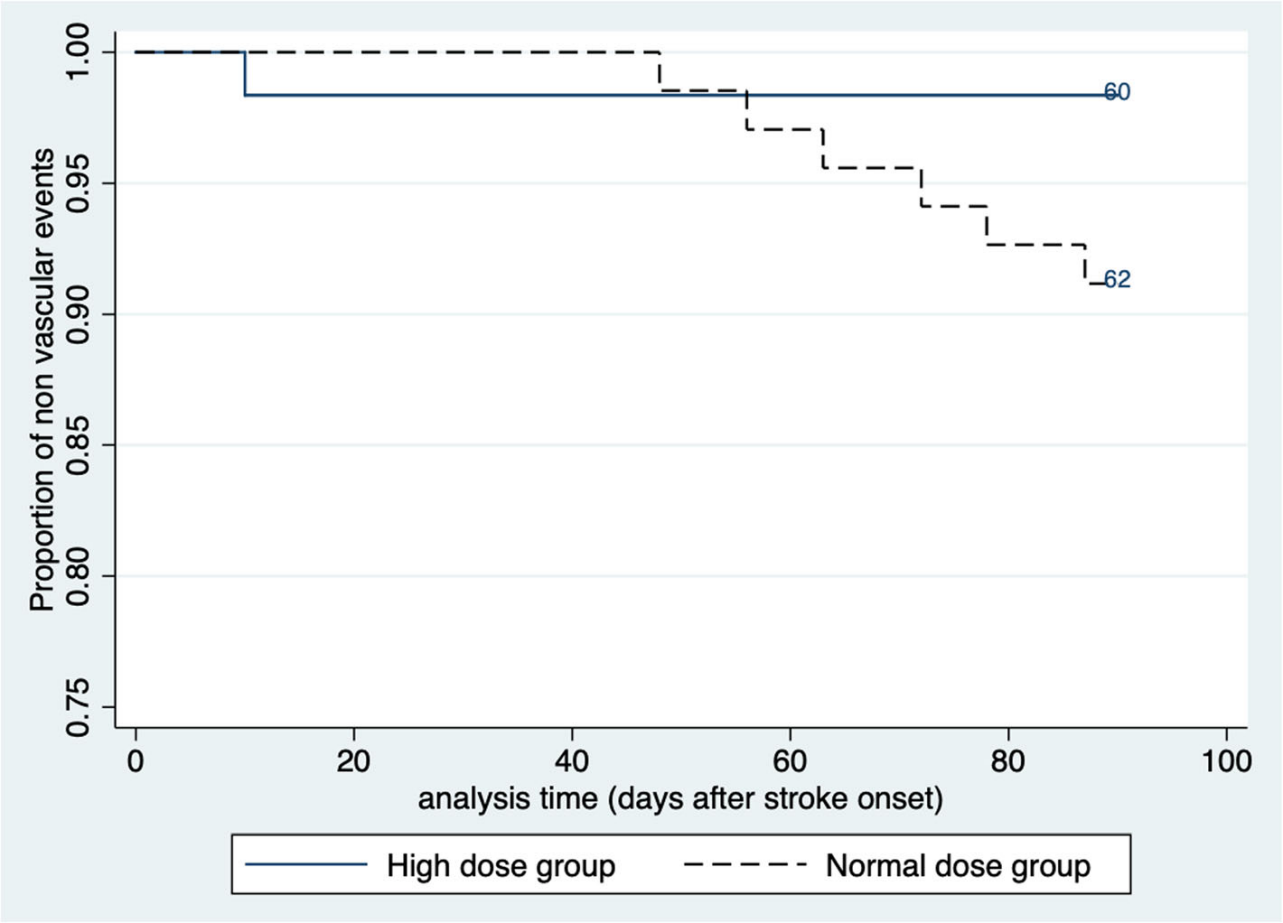
Kaplan-Meier survival estimates

The NIHSS scores at admission and discharge were not significantly different between the two groups. (Fig. 2). Of the 131 patients who completed the 90-day follow-up, seven had vascular events. Of these, one was in the high dose group (1.64%), and six were in the normal dose group (8.82%). The Kaplan-Meier survival estimates are shown in Fig. 3. In the log-rank test, the two groups were not significantly different from each other (*p* = 0.0763). The risk of vascular events within 90 days was not significantly different between the two groups in the Cox regression analysis (Table 2). Considering that the two groups differed in the prevalence of patients with history of diabetes, we added a history of diabetes to the adjustment and the risk of vascular events remained insignificantly different between the two groups.

**Table 2:** Effect of high dose clopidogrel versus normal dose clopidogrel on vascular events

	Hazard Ratio	95% Conf. Interval	*p*-value
Different groups	5.482	0.660–45.543	0.115
Adjusted by diabetes mellitus history			
Different groups	5.001	0.595–42.177	0.139
Diabetes mellitus	1.746	0.387–7.887	0.469

One patient in the normal dose group died of recurrent cerebral infarction within 90 days, and one patient in the high dose group was found to have subcutaneous haemorrhage. Ischaemic cerebrovascular disease recurred in one patient in the high dose group compared to three patients in the normal dose group, and angina was seen in two patients in the normal dose group. Patients in both groups did not experience intracranial haemorrhage, gastrointestinal haemorrhage, haemoptysis, pericardial occlusion, or other bleeding events leading to anaemia.

## Discussion

In this study, we enrolled patients with a minor acute ischaemic stroke within less than 7 days of onset (NIHSS ≤5) and moderate to severe stenosis of the cerebral blood supply arteries. Based on previous findings, these patients may benefit from dual antiplatelet aggregation therapy [1–4]. However, drug resistance may affect treatment outcomes. Activity of drug metabolism gene, *CYP2C19*, is an important factor in treatment outcomes. The selected intermediate metabolic gene carriers (*1/*2, *1/*3) in this study may not respond adequately to normal doses of clopidogrel [5], and increased doses may result in better outcomes [13, 14]. In this study, the high dose group did have fewer patients with vascular events within 90 days than the normal dose group, but this difference was not statistically significant.

Studies on the antiplatelet aggregation function and clinical effects of high-dose clopidogrel in *CYP2C19* genotype patients receiving PCI have presented conflicting results. It was found that a higher dose of clopidogrel results in better platelet inhibitory effects in patients with one LoFA, but patients with two or more LoFAs do not achieve the same degree of platelet inhibition [13, 16, 17]. In addition, other studies have found that increased doses of clopidogrel do not lead to better results [18, 19]. In the ARCTIC-GENE study [18], the investigators did not distinguish between homogeneous and heterogeneous *CYP2C19* *2 as a possible reason why increasing the clopidogrel dose still did not improve outcomes. In the GRAVITAS randomised trial [19], increasing the clopidogrel dose in patients with high treatment responsiveness as tested by VerifyNow P2Y12, did not clinically improve results compared to the standard-dose treatment group, but there were no more cases of bleeding. Metabolic genes were not taken into account in this study, and the fact that patients carrying LoFAs may not achieve improved clinical outcomes even with increased doses may be a possible reason for the negative trial results. An analysis of the above studies found that patients with intermediate metabolic patterns (only one LoFA) may benefit from clopidogrel dosing.

Studies with adjusted clopidogrel dosage or administration of an alternative drug by measuring platelet aggregation function or rate of inhibition (vasodilator stimulated phosphoprotein phosphorylation assay [VASP] or verifyNOW) have not demonstrated efficacy of therapeutic adjustment [20–22]. In addition, the existing methods for measuring platelet aggregation function include VASP, multiplate impedance aggregation (MP) and light transmission aggregation (LTA), etc. However, the lack of consistency between different platelet function tests makes it difficult to choose the appropriate test method [23]. Therefore, platelet function testing was not performed for each patient in this study.

Few studies have addressed the dose adjustment of clopidogrel for ischaemic stroke. Some investigators have found that administering 300 mg clopidogrel [24, 25] or 375 mg clopidogrel [26] for the first dose to patients with ischaemic stroke does not add to the bleeding risk. One study found [25] that administration of a loading dose improved neurological function in cerebral infarction patients 3 months after onset. A double maintenance dose of clopidogrel has not been studied in patients with ischaemic stroke. We excluded patients with normal metabolism of *CYP2C19* (*1/*1) to avoid an increased bleeding risk. In addition, we excluded patients with two LoFAs based on previous studies [13, 16, 17] to reduce the fact that increasing the dose did not increase the efficacy. There were no serious bleeding events in this study, suggesting that it may be safe to increase the dose of clopidogrel in this group of patients. In addition, the high dose group had fewer patients with vascular events within 3 months than the normal dose group, but the results did not reach statistical significance.

Other researchers have investigated alternatives to clopidogrel. A Japanese study found that prasugrel and clopidogrel were equally effective at preventing vascular events in non-cardioembolic stroke patients [27, 28]. A Chinese study found that ticagrelor plus aspirin treated patients with transient ischaemic attack (TIA) or minor stroke had lower platelet activity than those treated with clopidogrel, especially *CYP2C19* LoFA carriers [29]. Clopidogrel is more economical and accessible than the two drugs mentioned above, and if better efficacy is obtained in screened patients by increasing the dose, better societal benefits can be obtained.

In this study, the two groups had different proportions of patients with a history of diabetes, which may have influenced the final results. Diabetes may affect the response to clopidogrel in patients with cerebrovascular disease. There are many reasons for poor responsiveness to clopidogrel in patients with diabetes, including metabolic, genetic, and other clinical factors [30–32]. However, it has also been shown that clopidogrel has a consistent effect on platelet inhibition in both diabetic and non-diabetic patients [33]. In a study of metabolic genes, diabetic CYP2C19*2 carriers were found to have a poorer response to clopidogrel than diabetic non-carriers, while non-diabetics showed no such difference [34]. In another study, both CYP2C19*2 carrier and non-carrier diabetic patients had poorer responses to clopidogrel than non-diabetic patients, but there was no difference between the two, and non-diabetic CYP2C19*2 carriers had higher platelet reactivity than non-carriers after clopidogrel application [35]. In the present study, when adjusted for diabetes, the results did not change.

There are several limitations in this study. First of all, this is an open-label study in only one academic stroke centre, and the results should be carefully interpreted. Second, the patients and investigators were not blinded, which may have introduced bias in the outcome assessments. Third, the sample size was small, and the proportion of patients with diabetes was different between the two groups, indicating that the basic characteristics of the two groups of patients were not completely consistent, which may have affected the results. We adjusted for diabetes in the final comparison of the results. Despite these limitations, the absence of serious bleeding events in our study suggests that larger and more rigorous clinical trials may be attempted to understand the effectiveness and safety of high dose clopidogrel therapy.

## Conclusion

In patients with ischaemic stroke who had only one *CYP2C19* LoFA and moderate to severe cerebral stenosis, fewer vascular events occurred within 3 months with high dose clopidogrel combined with aspirin than with normal dose clopidogrel combined with aspirin. However, no significant difference was observed between the two groups. In addition, neither the high nor the normal dose group experienced bleeding events.

## Data Availability

The datasets used and/or analysed during the current study are available from the corresponding author upon reasonable request.

## Abbreviations

SAMMPRIS: Stenting and Aggressive Medical management for prevention of Recurrent Stroke in Intracranial Stenosis trial
WASID: Warfarin Aspirin Symptomatic Intracranial Disease trial
TCD: Transcranial Doppler
LoFA: Loss-of-function allele
PCI: Percutaneous coronary intervention
NIHSS: National Institutes of Health Stroke Scale
MRS: Modified Rankin Scale
TIA: Transient ischaemic attack
CYP: Cytochrome P-450
